# Expansion and characterization of epithelial stem cells with potential for cyclical hair regeneration

**DOI:** 10.1038/s41598-020-80624-3

**Published:** 2021-02-10

**Authors:** Makoto Takeo, Kyosuke Asakawa, Koh-ei Toyoshima, Miho Ogawa, JingJing Tong, Tarou Irié, Masayuki Yanagisawa, Akio Sato, Takashi Tsuji

**Affiliations:** 1 Laboratory for Organ Regeneration, RIKEN Center for Developmental Biology (CDB) and RIKEN Center for Biosystems Dynamics Research (BDR), Hyogo, 650-0047 Japan; 2 Organ Technologies Inc., Tokyo, 101-0048 Japan; 3 Department of Bioscience, Graduate School of Science and Technology, Kwansei-Gakuin University, Hyogo, 669-1337 Japan; 4 Division of Anatomical and Cellular Pathology, Department of Pathology, Iwate Medical University, Iwate, 028-3694 Japan; 5 Department of Plastic and Aesthetic Surgery, School of Medicine, Kitasato University, Kanagawa, 252-0375 Japan; 6 Department of Plastic and Reconstructive Surgery, School of Medicine, Keio University, Tokyo, 160-8582 Japan

**Keywords:** Adult stem cells, Regeneration, Skin stem cells

## Abstract

In mammals, organ induction occurs only during embryonic development except for hair follicles (HFs). However, HF-resident epithelial stem cells (HFSCs), which are responsible for repetitive HF regeneration, are not fully characterized. Here, we establish in vitro culture systems that are capable of controlling the ability of HFSCs to regenerate HFs. Based on a method that precisely controlled the number of HFs for regeneration, functional analysis revealed that CD34/CD49f/integrin β5 (Itgβ5)-triple-positive (CD34+/CD49f+/Itgβ5+) cells have multipotency and functional significance for continual hair regeneration. In native HFs, these cells reside in the uppermost area of the bulge region, which is surrounded by tenascin in mice and humans. This study unveils the subpopulation of HFSCs responsible for long-term hair cycling of HFs regenerated from bioengineered HF germ, suggesting the presence of functional heterogeneity among bulge HFSCs and the utility of our culture system to achieve HF regenerative therapy.

## Introduction

Organogenesis occurs only during embryonic development via complex processes involving tissue self-organization, cell–cell interactions, the regulation of signaling molecules, and cell motility in each organ-forming field according to the embryonic body plan. During embryonic development, most organs arise from organ germs, which are induced by reciprocal interactions between epithelial and mesenchymal stem cells with organ-inductive potential; these cells lose their potential after birth. By mimicking embryonic organogenesis, we previously proved the concept of fully functional regeneration of ectodermal organs, including hair follicles (HFs), from embryonic organ-inductive potential stem cells^1–6^. Furthermore, the successful generation of organoids, which partially recapitulate the structure and function of their original organ and are derived from multiple types of adult tissue stem cells, has demonstrated the potential of these cells for organ induction, at least in part^7^. However, functional regeneration of an entire organ utilizing adult tissue stem cells has not been achieved, except with HFs^1–6^.

**Figure 1:**
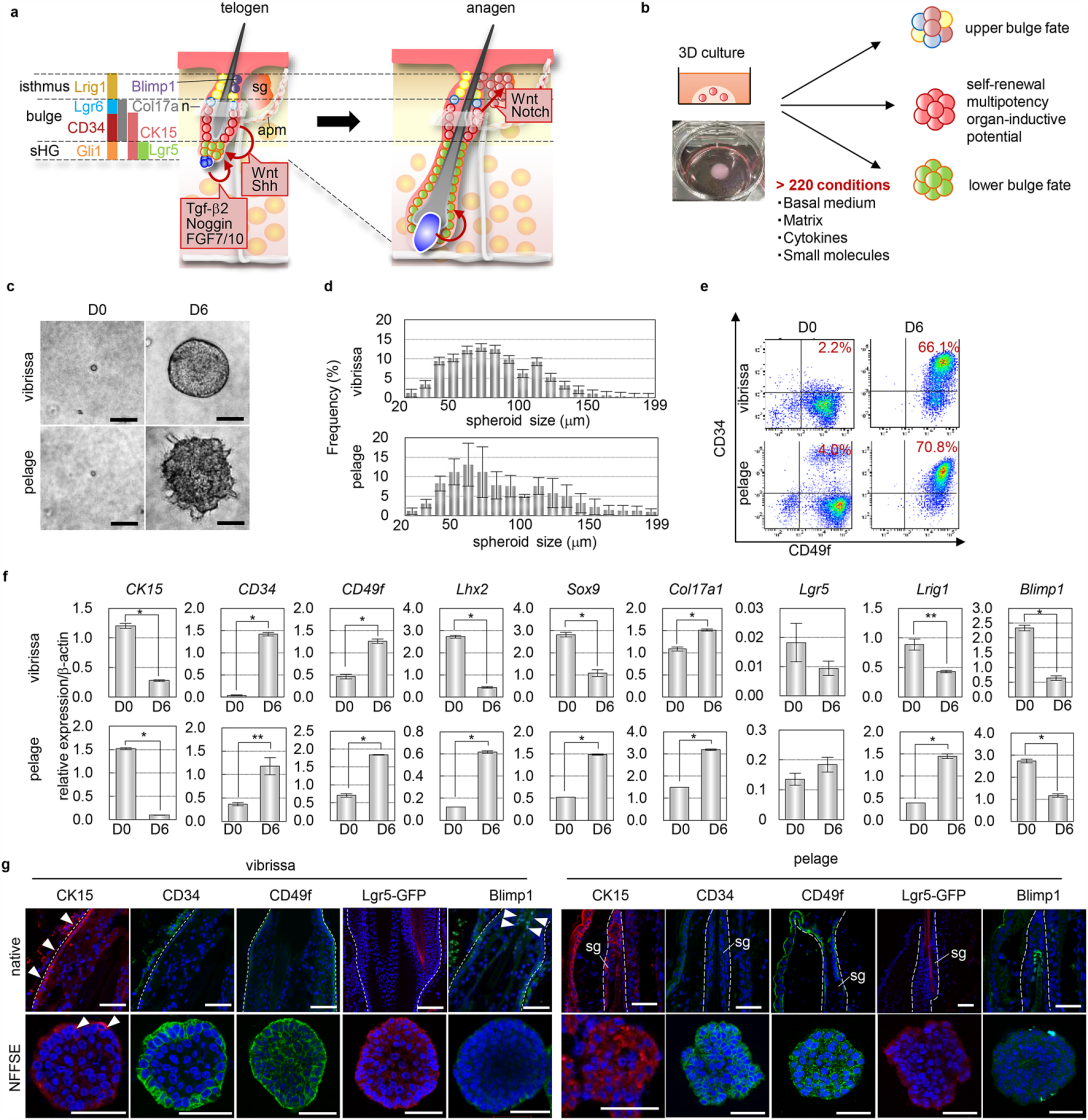
Identification of culture conditions that can control the status of HFCSs. (**a**) Schematic illustration of reported HFSCs in mouse pelage HF and signaling pathways contributing to HFCS maintenance and activation. (**b**) Experimental scheme of the 3D cell culture for HF bulge epithelial cells. Epithelial cells were isolated by micro dissection and subsequent enzyme treatment from the bulge of adult mouse vibrissa at the growing phase, anagen, or back skin at the resting phase, telogen, including pelage HFs. (**c**) Phase contrast images of cultured cells isolated from adult mouse vibrissa (upper panels) or pelage HF (lower panels) at the indicated time point in 3D culture. (**d**) Quantification analysis of the size of spheroids cultured in NFFSE medium. Frequency was calculated from 1,000 spheroids (**e**) Replicative FACS plot for indicated markers on freshly isolated and cultured epidermal cells. (**f**) Replicative qPCR analysis of indicated markers on freshly isolated or cultured cells. (**g**) Representative immunofluorescence of the indicated markers on native vibrissa HFs, pelage HFs, and cultured spheroids of C57BL/6 or Lgr5-EGFP mice. Dashed lines indicate the border between the epithelium and dermis. sg, sebaceous gland. Data are presented as the mean ± SD. *p < 0.001; **p < 0.002. Scale bars, 20 μm. See also Supplemental Fig. S1.

The most fascinating characteristic of hair follicles is their ability to cyclically undergo follicular regeneration after birth. The HF of mammals is largely divided into two major regions: a permanent upper portion containing the bulge region, infundibulum, isthmus and sebaceous gland; and a variable lower portion including hair matrix, where the hair shaft is generated, and differentiated epithelial cells. Throughout an individual’s life, the variable lower portion undergoes cyclical periods of growth (anagen) and quiescence (telogen) which compose the hair cycle. This process is driven by reciprocal interactions between HFSCs, which reside in the cytokeratin 15 (CK15)- or CD34-positive bulge and the Lgr5-positive secondary hair germ (sHG), and mesenchymal dermal papilla cells (DPCs)^8,9^ (Fig. 1a). HFs also harbor multiple types of fate-restricted stem cells responsible for maintaining the upper portion of the HF; these cells express proteins such as Lgr6, Lrig1, and Blimp1^10–12^ (Fig. 1a). Among HFSCs, several stem cells, such as CK15-, Lgr5-, Lgr6-, and Lrig1-expressing cells, show plasticity and have the ability to regenerate entire HFs following full-thickness wounds as well as within hair reconstitution assays^9–11,13^. Therefore, adult HFSCs could be a promising candidate for organ regenerative medicine, and a method to efficiently expand HFSCs that have the ability to sustainably induce HF regeneration is required. However, whether functional heterogeneity exists and, if so, what types of stem cells have the ability to sustain long-term hair cycles are unclear, although single-cell analysis clearly demonstrated heterogeneity among cell populations expressing the same stem cell marker, such as CD34 and CD49f double-positive cell populations (CD34+/CD49f+), a well-established marker for bulge HFSCs^14^.

The molecular mechanisms regulating the maintenance, proliferation and differentiation of HFSCs have been extensively characterized via genetic gain- and loss-of-function studies^8,13,15–20^. (Fig. 1a). Most strikingly, Wnt activation concomitant with BMP suppression initiates the activation of HFSCs, and subsequent Shh activation specifies the fate of the progeny of HFSCs to the lower HF region. On the other hand, activation of Wnt and Notch signaling is involved in the differentiation of the sebaceous gland from HFSCs. By mimicking these molecular mechanisms, a previous study demonstrated that CD34+/CD49f+ cells isolated from adult mouse back skin can be amplified fivefold in 14 days by 3D culture using Matrigel or type I collagen as a scaffold and medium containing EGF, FGF2, and VEGF-A^21,22^. Moreover, this pioneering study demonstrates the de novo generation of HFSCs from non-HFSCs in a self-organized manner and opens the door to successful HF regenerative treatments. However, whether these cells contain the HFSCs responsible for cyclical hair regeneration is not clear.

Here, we identify a subpopulation of HFSCs capable of sustaining the long-term hair cycle of HFs regenerated from bioengineered HF germ. We established culture conditions that enabled us to control the differentiation status of mouse HFSCs. The comparison of cell populations that are cultured in different conditions revealed that among the CD34 and CD49f double-positive cell populations (CD34+/CD49f+), integrin β5 (Itgβ5)-positive cells were enriched in the cell cultures. Withdrawal of CD34+/CD49f+/Itgβ5+ cells from bioengineered HF germ results in limited periods of hair cycles. In native hair follicles, CD34+/CD49f+/Itgβ5+ triple-positive cells were found in the uppermost area of the bulge region surrounding tenascin in both mice and humans. Collectively, our results indicate that CD34+/CD49f+/Itgβ5+ cells compose the key cell population required for sustainable hair cycles of regenerated HF from bioengineered HF germ, and the culture system established in the current study provides a platform for harvesting HFSCs in practical use for HF regenerative therapy.

## Results

### Establishment of a 3D culture system to efficiently expand CD34/CD49f double-positive bulge cells

In the past decade, many stem cells with organ-inductive potential have been identified utilizing a novel 3D culture system, which allows organ-inductive stem cells to generate organoids that include both the stem cells themselves and their microenvironmental niches in a self-organized manner^23^.

To identify HFSCs that are indispensable for long-term cyclical HF regeneration, we optimized the culture conditions under which HFSCs can maintain or lose their capacity to cyclically induce hair regeneration and then compared the cell populations that were cultured in these different conditions. Because we targeted all cells in the bulge region, including not only HFSCs but also their niche cells, we isolated all cells in the bulge region from vibrissa HFs in various stages of the hair cycle by microdissection and subsequent enzyme treatment. We examined more than 220 combinations of extracellular matrix (ECM) and previously defined cytokine insights into the proliferation and maintenance of HFSCs^8,13,15–21^ (Fig. 1a,b). On day 6 in culture, single epithelial cells subjected to 3D culture with Noggin, FGF-7, FGF-10, sonic hedgehog agonist, and EGF (NFFSE medium) and with atelocollagen, the defined type I collagen that lacks telopeptide regions and thus can be used for clinical application (unlike cancer-derived Matrigel), formed round-shaped spheroids of varying sizes with the highest spheroid-forming efficiency at 32.0 ± 9.8% and an amplification rate of 198.0 ± 22.3-fold (Fig. 1b,d, Supplemental Fig. S1a). To examine whether pelage epithelial cells can also expand in these conditions, we cultured epithelial cells isolated from telogen back skin, including interfollicular epithelial cells and pelage HFSCs. Similar to the vibrissa bulge cells, single back skin epithelial cells give rise to spheroids with a rough surface at a spheroid-forming efficiency of 23.0 ± 9.8% and an amplification rate of 7.3 ± 2.6-fold (Fig. 1c,d). The difference between vibrissa bulge cells and back skin cells may be due to the percentage and hair cycle stage of HFSCs in each cell population. NFFSE conditions drastically increased the proportion of CD34+/CD49f+ cells by 66.1% for vibrissa and 70.8% for back skin cells (Fig. 1e). Consistent with this, gene expression analysis by real-time quantitative PCR (qPCR) revealed upregulation in the expression of *CD34*, *CD49f* and type XVII collagen (*Col17a1*), a marker for bulge HFSCs involved in their maintenance^24^, in both vibrissa and back skin cells, while no significant upregulation was observed for *Lgr5*, a marker for stem cells committed to lower pelage HF fate, or for *Blimp1*, a marker of upper HF cells and sebaceous gland stem cells (Fig. 1f, Supplemental Fig. S1b). The expression changes of other conventional stem cell markers, including *Lhx2* and *Sox9*, varied depending on the cell source, possibly because of the difference in cell types and hair cycle stage. Immunohistochemical analysis of individual spheroids further confirmed the qPCR data in both vibrissa- and pelage-derived cells (Fig. 1g). These data indicate that 3D culture utilizing atelocollagen together with medium containing Noggin, FGF7, FGF10, SAG, and EGF shows an improved amplification rate of CD34+/CD49f+ cells and a shorter culture period required for the expansion of CD34+/CD49f+ cells compared to that of a previously reported method^21^.

### Bulge cells cultured in NFFSE medium show multipotency in vitro

To examine whether cells cultured in NFFSE medium maintained their undifferentiated status and multipotency, we further developed our culture conditions, which allowed us to modify the fate of HFSCs.

**Figure 2:**
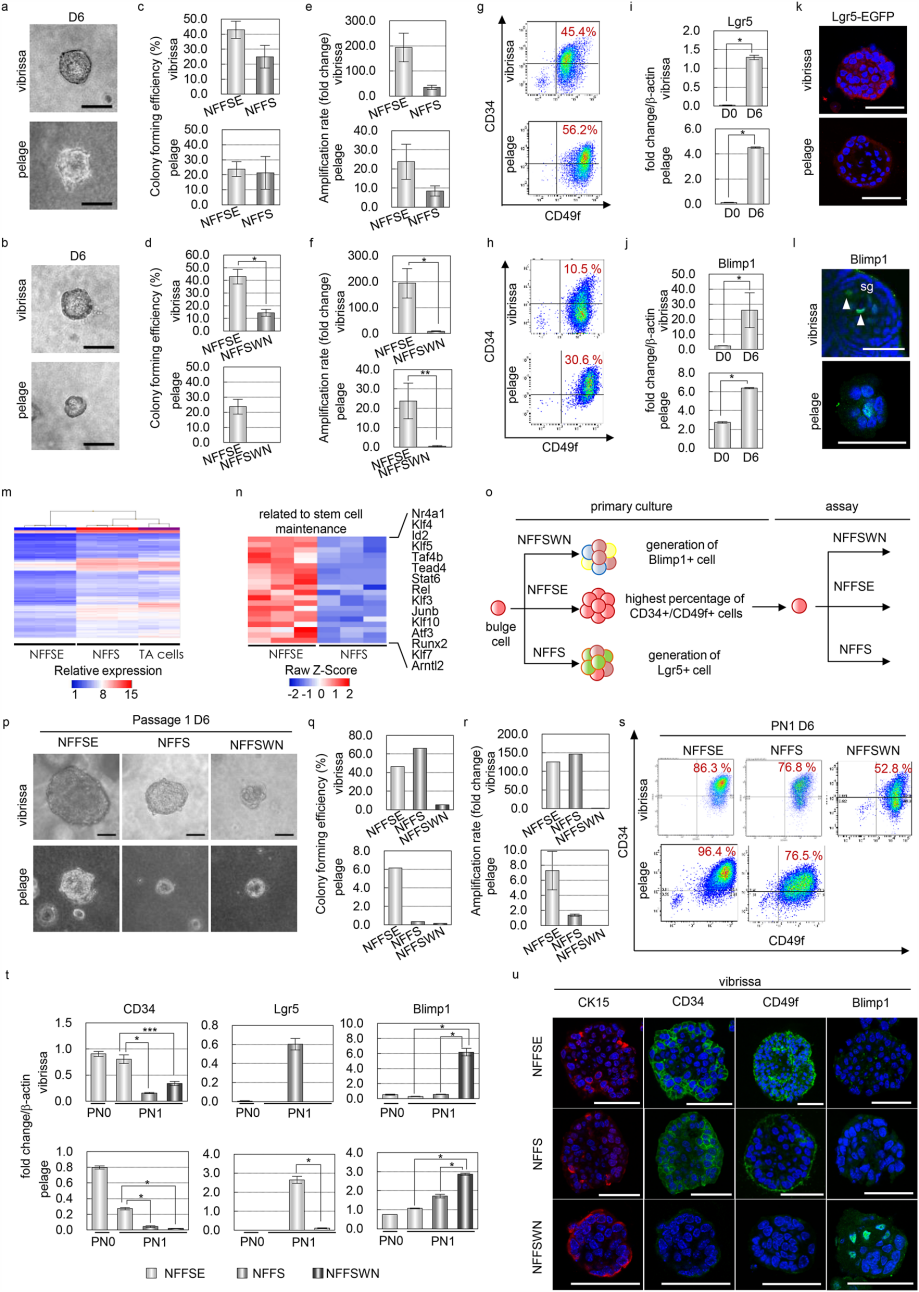
NFFSE cultured cells show multipotency in vitro*.* (**a**,**b**) Phase contrast images of spheroids cultured for 6 says in NFFS (**a**) and NFFSWN medium (**b**). (**c**,**d**) Colony forming efficiency of cultured cells in the indicated medium. (**e**,**f**) Amplification rate of cultured cells in the indicated medium. (**g**,**h**) Replicative FACS plot of NFFS- (**g**) and NFFSWN-cultured epidermal cells (**h**). (**i**,**j**) qPCR analysis of the indicated markers on freshly isolated or cultured cells. (**k**,**l**) Representative immunofluorescence of the indicated markers on cultured spheroids. (**m**) Clustering analysis of array data obtained from NFFSE- and NFFS-cultured cells and TA cells of hair follicle. (**n**) Heatmap of selected transcription factors related to stem cell maintenance, which are differentially expressed between NFFSE and NFFS conditions in microarray analysis. (**o**) Experimental scheme of in vitro differentiation analysis. Epithelial cells isolated from the bulge of adult mouse vibrissa or back skin, including pelage HFs, were cultured in NFFSE medium for 6 days and then passed into NFFSE, NFFS, or NFFSWN medium. (**p**) Phase contrast images of cultured vibrissa and pelage HFs at day 6. (**q**) Colony forming efficiency in the indicated culture medium at day 6 following passage. (**r**) Amplification rate following passage in indicated medium. (**s**) Replicative FACS plot of cultured cells after passage in the indicated medium. (**t**) Replicative qPCR analysis of indicated markers on cultured cells. (**u**) Representative immunofluorescence of the indicated markers on spheroids of C57BL/6 or Lgr5-EGFP mice cultured in the indicated conditions. Arrowheads indicate the expression of the indicated marker. Data are presented as the mean ± SD. *p < 0.001; **p < 0.005; ****p < 0.002. Scale bars, 20 μm. See also Supplemental Fig. S2.

We found that withdrawal of EGF from NFFSE medium (NFFS medium) and the addition of Wnt ligand, Notch ligand, and TGF-β inhibitor (NFFSWN medium) resulted in a decrease in colony-forming efficiency, colony size, amplification rate, and percentage of CD34/CD49f-double-positive (CD34+/CD49f+) cells (Fig. 2a,j, Supplemental Fig. S1a). Importantly, NFFS- and NFFSWN-cultured cells expressed high levels of Lgr5 or Blimp1, suggesting the commitment of these cells to lower and upper HF fates, respectively (Fig. 2k,l, Supplemental Figs. S1b and S2a,b). Microarray and subsequent clustering analysis with a previously published gene expression profile of HF transient amplifying (TA) cells revealed that the gene expression profile of NFFS-cultured vibrissa cells is similar to that of TA cells^25^ (Fig. 2m). Moreover, we found that NFFSE-cultured cells highly expressed numerous transcription factors involved in stem cell maintenance, including *Klf4*^26,27^, compared to cells cultured in NFFS medium (Fig. 2n).

To examine the multipotency of NFFSE-cultured cells, we passaged them into either NFFSE medium or differentiation medium (Fig. 2o); we found that NFFSE-cultured cells showed decreases in the colony size and CD34+/CD49f+ cell population and increases in the expression of committed stem cell markers, such as Lgr5 or Blimp1, at the gene and protein expression levels in response to NFFS and NFFSWN conditions, similar to the changes observed in primary culture (Fig. 2p–u, Fig. S2c–g). Taken together, these results suggest that HFSCs maintain a relatively undifferentiated status in NFFSE medium.

### NFFSE-cultured cells have the ability to cyclically induce hair regeneration

To examine whether these cultured cells possess the ability to cyclically induce hair regeneration, we performed a hair reconstitution assay by using the organ germ method, which mimics organ germ formation during organogenesis by spatially arranging organ-inductive epithelial stem cells and mesenchymal stem cells in collagen gel^3^. Compared to conventional hair reconstitution assays, such as patch and chamber assays, this assay system has several advantages, including fewer cells required for hair reconstitution, high efficiency of hair regeneration per number of cells used, and the ability to control the number of regenerating HFs, which allows us to analyze the number of hair cycles of an individual regenerated hair^3^.

**Figure 3:**
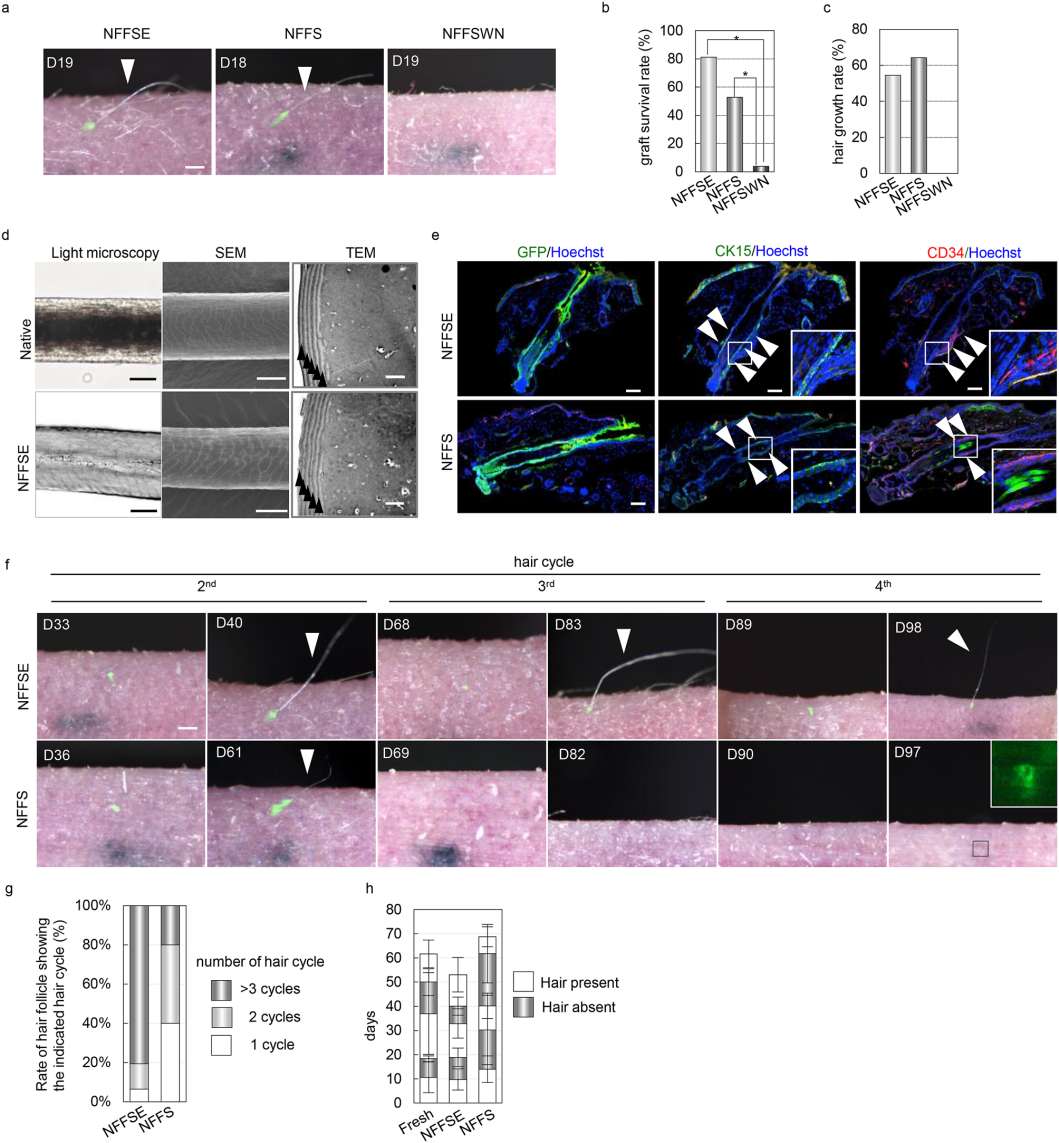
NFFSE cultured cells have the ability to undergo repetitive HF regeneration. (**a**) Macro-morphological observations of transplanted bioengineered HF germ generated from vibrissa bulge cells cultured in the indicated medium. Bioengineered HF germ generated from cultured HF epithelial cells and dermal papilla cells, which were isolated from adult vibrissa HFs, was transplanted into back skin of nude mice intracutaneously. (**b**) Quantification of graft survival rate, (**c**) Quantification of first hair growth rate after transplantation. (**d**) Microscopic observation of the regenerated hair shafts by light microscopy, scanning electron microscopy (SEM), and transmission electron microscopy (TEM). (**e**) Representative immunofluorescence of the indicated markers on regenerated HFs. (**f**) Long-term hair cycle analysis of regenerated HFs. (**g**) Quantification of the percentage of regenerated HFs showing indicated number of the hair cycle (**s**). (**h**) Quantification of the duration of days with or without hair shaft in each hair cycle. Arrowheads indicate the regenerated hair shaft in (**a**,**f**), cuticle layers in (**d**), and expression of indicated markers in (**e**). Data are presented as the mean ± SD. *p < 0.01. Scale bars, 100 μm in (**a**,**f**); 20 μm in the left and middle panel and 2 μm in the right panel of (**d**); 20 μm in (**e**).

We purposefully generated bioengineered vibrissa HF germs from which only a few hairs regenerate, utilizing cultured HF epithelial cells isolated from CAG-EGFP mice and cultured DPCs. By 3 weeks after transplantation of bioengineered germ generated from NFFSE-, NFFS-, and NFFSWN-cultured cells, the eruption and growth of hair shafts were observed at frequencies of 54.5% (n = 155), 61.2% (n = 92) and 0% (n = 78), respectively (Fig. 3a–c). Regenerated hair shafts showed the correct morphological and histological structure, including the hair cortex and 6 layers of thin cuticles under a light microscope and an electron microscope^3^ (Fig. 3d). Immunohistochemical analysis of regenerated HFs at the 2nd hair cycle from both NFFSE- and NFFS-cultured cells demonstrated the presence of cells expressing common HFSC markers, including CD34 and CK15, in the bulge region (Fig. 3e). Long-term analysis revealed that 81.0% of HFs regenerated from NFFSE-cultured cells showed repetitive hair cycles of at least 3 cycles, with similar hair cycle duration to that of HFs regenerated from freshly isolated bulge epithelial cells (Fig. 3g, h). In clear contrast, 79.0% of regenerated HFs from NFFS-cultured cells showed fewer than 2 cycles of hair regeneration (p = 0.002, Student’s t-test), indicating the depletion of HFSCs, which is required for cyclical hair regeneration, in NFFS culture conditions compared to NFFSE conditions. These results suggest that cells cultured in NFFSE conditions were rich in HFSCs possessing the potential to cyclically induce hair regeneration, a hair cycle, in addition to the self-renewing capacity.

### CD34+/CD49f+/Itgβ5+ cells are indispensable for cyclical hair regeneration

Stem cell self-renewal is tightly regulated by both intrinsic and extrinsic cues driven by spatially distinct microenvironments, termed niches, and cell adhesion molecules and ECM have an important role in maintaining multiple stem cell lines, including hematopoietic and mesenchymal stem cells^10–12,24,28–30^.

**Figure 4:**
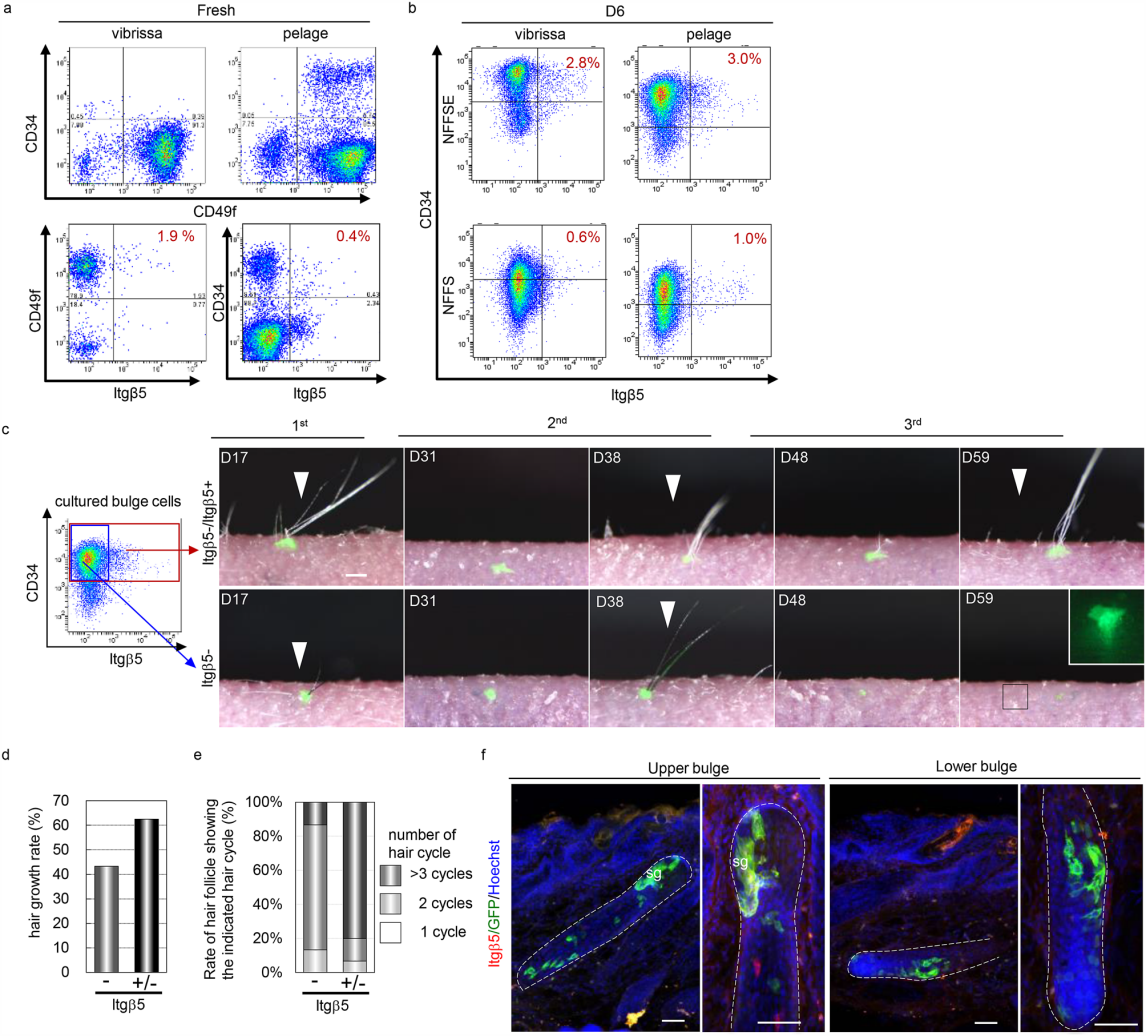
Itgβ5+ cells are required for repetitive HF regeneration. (**a**,**b**) Replicative FACS plot of freshly isolated (**a**) and cultured cells (**b**) for the indicated markers. (**c**) Long-term macro-morphological observations of transplanted bioengineered HF germ generated from CD34+/CD49f+/Itgβ5-cells (lower panel) and CD34+/CD49f+/Itgβ5-cells mixed with CD34+/CD49f+/Itgβ5+ cells (upper panel). (**d**) Quantification of first hair growth rate after transplantation. (**e**) Quantification of the percentage of regenerated HFs showing indicated number of the hair cycle (**s**). (**f**) Representative immunofluorescence of the indicated markers on regenerated HFs. CD34+/CD49f+/Itgβ5+ cells isolated from NFSSE cultured pelage epithelial cells of CAG-EGFP mice were combined with freshly isolated skin epithelial cells isolated from ED18 mouse embryos at a 1:9 ratio and processed to generate bioengineered organ germ using embryonic dermal cells. Right panels indicate a high magnification of the boxed area in the left panels. Arrowheads indicate the regenerated hair shaft. sg, sebaceous gland. Data are presented as the mean ± SD. Scale bars, 50 μm in (**g**); 100 μm in (**d**). See also supplemental Fig. S3.

FACS analysis for more than 20 cell surface markers showed that cells cultured in NFFSE medium were enriched with CD34+/CD49f+/Itgβ5+ cells, which were also present in freshly isolated bulge cells of pelage HF (Fig. 4a,b Fig. S3). Although the bulge region of vibrissa HF expressed low levels of CD34, CD49f+ basal cells expressing high levels of Itgβ5 were present (Fig. 4a). To examine the functional significance of CD34+/CD49f+ /Itgβ5+ cells, we took advantage of a hair reconstitution assay, which can reliably eliminate the target cells. We performed a hair reconstitution assay utilizing NFFSE-cultured pelage epithelial cells following FACS isolation and found that CD34+/CD49f+/Itgβ5- cells and mixed populations with CD34+/CD49f+/Itgβ5+ cells yielded hair regeneration at frequencies of 43.2% (n = 57) and 62.5% (n = 48), respectively (Fig. 4c,d). Strikingly, the percentage of regenerated HFs showing at least 3 cycles of hair cycle drastically increased from 13.3 to 79.9% (p = 0.002, Student’s t-test) when Itgβ5+ cells were present in the bioengineered HF germ (Fig. 4c,e). In addition, we performed a competition hair reconstitution assay using CD34+/CD49f+/Itgβ5+ cells isolated by FACS from NFSSE-cultured pelage epithelial cells from CAG-EGFP mice and skin epithelial cells isolated from embryonic day (ED) 18 mouse embryos by mixing at a 1:9 ratio. Histological analysis of regenerated HFs revealed that GFP-positive cells gave rise to multiple cell lineages composing HFs, including sebaceous glands, bulges, and hair bulbs (Fig. 4f). Taken together, these results clearly demonstrated that Itgβ5+ cells among the CD34+/CD49f+ cell population are multipotent and are vital for long-term cyclical induction of hair regeneration.

### Itgβ5+ cells are located in the upper bulge region surrounded by tenascin in native hair follicles

**Figure 5:**
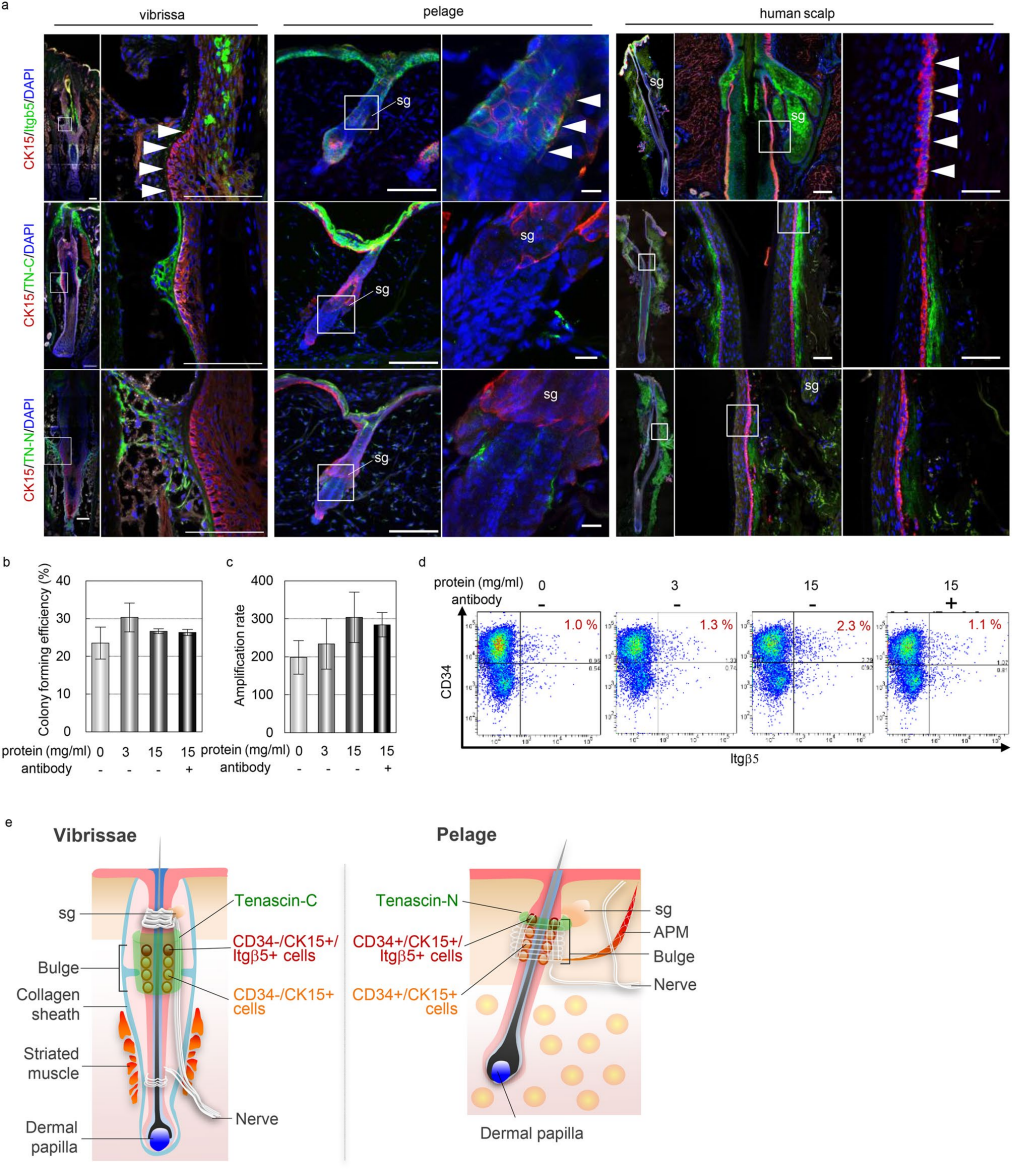
Itgβ5+ cells locate in the upper bulge region surrounding tenascin in native hair follicle of mouse and human. (**a**) Immunofluorescence of the indicated markers on native mouse vibrissa, pelage, and human scalp HF. Right panels indicate a high magnification of the boxed area in the left panels. (**b**) Colony forming efficiency of cultured vibrissa bulge cells in the indicated conditions with recombinant TN-C proteins and TN-C neutralizing antibody. (**c**) Amplification rate of cultured vibrissa bulge cells in the indicated conditions. (**d**) Replicative FACS plot of cultured vibrissa bulge cells. (**e**) Schematic representation of the localization of Itgβ5+ cells, CK15 positive cells, and tenascin-C/N expression in native vibrissa and pelage hair follicle. Arrowheads indicate the expression of the indicated markers. APM, arrector pili muscle. sg, sebaceous gland. Data are presented as the mean ± SD. Scale bars, 50 μm. See also supplemental Figs. S4 and S5.

HFs harbor multiple types of fate-restricted stem cells in individual niches^10–12^. Although previous studies indicated that a specialized HF niche is associated with the expression of distinctive ECM proteins, such as tenascins and cadherins, the functional significance of the ECM is largely unknown except for Col17a and nephronectin^24,29,30^. To examine whether CD34+/CD49f+/Itgβ5+ cells are present in native HFs and, if so, what is the niche for these cells, we performed an immunohistochemical analysis for Itgβ5 and a variety of cell surface receptors and ECM proteins that potentially bound to integrins (Fig. 5a). In mouse vibrissa HFs, Itgβ5+ cells are located in the upper part of the bulge region and express CK15 (Fig. 5a and Supplemental Fig. S4b). We observed the innervation of Tuj-1 + nerve cells, and the expression of Gli1, a component of Shh signaling, was expressed in the lower part of the Itgβ5+ region (Supplemental Fig. S4b). Moreover, we found that the upper bulge region, where Itgβ5+ cells are present, was surrounded by tenascin-C (TN-C) (Fig. 5a,e). In mouse pelage HFs, similar to vibrissa HFs, Itgβ5+ cells were observed in CK15-expressing upper bulge regions, where innervation and Gli1 expression were also observed (Fig. 5a and Supplemental Fig. S4b). In contrast to vibrissa HFs, Itgβ5+ cells in pelage HFs were found in the upper bulge region defined by the expression of tenascin-N (TN-N) (Fig. 5a,e). In human scalp HFs, Itgβ5+ cells were found in the region associated with the ECM expressing both TN-C and TN-N (Fig. 5a). To examine the functional significance of tenascin in Itgβ5+ cells, we performed a cell culture analysis with recombinant TN-C protein and a neutralizing antibody and found that although there was no statistically significant difference, the percentage of CD34+/CD49f+/Itgβ5+ cells at 6 days in culture tended to increase with increasing doses of TN-C. This effect was neutralized by the addition of TN-C antibody, while the colony-forming efficiency, late amplification, and colony size were not affected (Fig. 4b–d, Supplemental Fig. S5a,b). Although the function and the causal relationship of tenascins and Itgβ5+ cells need to be examined in vivo, these results suggest the presence of heterogeneous subpopulations in CK15-expressing bulge stem cells as well as the possibility that Itgβ5+ cells have a role in sustaining the long-term hair cycle in vivo. Furthermore, the upper part of the bulge defined by the expression of tenascins is a possible niche for Itgβ5+ cells.

## Discussion

In this study, we unveiled a novel cell population expressing CD34/CD49f/Itgβ5 that contributes to long-term cyclical HF regeneration in a hair reconstitution assay by establishing multiple culture conditions that enable us to expand and control the differentiation status of HFSCs. Itgβ5+ cells are located in the upper most region of the bulge area, which is surrounded by tenascin-C and/or tenascin-N, in mouse vibrissa and pelage HFs as well as in human scalp HFs. These findings significantly advance our understanding of the mechanisms underlying the regulation of HFSCs and shed right on the possible presence of functional heterogeneity in the HFSC population, which expresses the same stem cell markers. Moreover, our findings will contribute to the goal of HF regenerative medicine.

Past studies demonstrated that mammalian HFs are maintained by a heterogeneous population of fate-restricted HFSCs and their niches^10–12^. Moreover, recent single-cell analysis revealed the presence of a heterogeneous population even among cells expressing the same stem cell marker, such as CD34^14^. However, this functional heterogeneity is not fully understood. In the natural HF in both mouse and human, Itgβ5+ cells are found in the upper most area of the bulge. Past studies using lineage-tracing approaches showed that a subset of stem cells expressing K15 or Lgr6 can migrate either downwards towards the progeny committed to the lower HF or upwards towards the sebaceous gland^31,32^. In this study, we found that CD34+/CD49f+/Itgβ5+ cells have the ability to induce cyclical hair regeneration in a hair reconstitution assay, indicating the presence of functional heterogeneity among cultured CD34+/CD49f+ cells. Based on these facts and our data, we speculate that Itgβ5+ cells found in the upper part of native HFs directly or indirectly play an important role in long-term cyclical hair regeneration.

Adult stem cells are maintained by both intrinsic and extrinsic mechanisms via specialized niches. In the bulge region in the vibrissa or pelage HF of mice, specialized ECM proteins, such as COL17A1 (a known essential mediator for the self-renewal and maintenance of HFSCs), are expressed^24^. In this study, we observed TN-C expression in the bulge region of mouse vibrissa as previously reported^30^. Moreover, we found that TN-C and/or TN-N are also expressed in the human scalp HF and mouse pelage HF. All tenascins share the same molecular architecture, such as an EGF-like domain^33^. Given that the polymerized TN-C domain is incapable of inducing EGF activation^34^, tenascin may keep Itgβ5+ HFSCs in a quiescent state by inhibiting EGF-mediated signaling. This finding may be consistent with our results in culture experiments showing that EGF is required for the expansion of CD34+/CD49f+/Itgβ5+ HFSCs, which are required for sustainable hair regeneration in hair reconstitution assays.

In mammals, organogenesis occurs only during embryonic development except in very restricted organs, including HFs. Although numerous pioneering studies have demonstrated the potential of induced pluripotent stem cells (iPSCs) and adult tissue stem cells to partially mimic the structure and function of their original organs, full functional regeneration of the entire organ was achieved only in HFs^1–6^. Therefore, HF regeneration could be a promising method for organ regenerative medicine. In past decades, much effort has been devoted to establishing a method to expand HFSCs while maintaining their organ-inductive ability. Recently, Chacón-Martínez and colleagues established a culture method to maintain the regenerative capacity even after 15 passages. In the present study, we found the condition to increase HFSC populations approximately 200-fold, which is much improved from previous studies. Moreover, the expression of Itgβ5 in addition to CD34/CD49f could be a good marker to evaluate the ability of cultured HFSCs to cyclically induce hair regeneration. Findings from Chacón-Martínez and colleagues as well as from our research group will significantly advance the technological development of HF regenerative therapy.

The function and mechanism of Itgβ5 and tenascins in stem cell maintenance and cell kinetics should be further studied. Nevertheless, the culture conditions identified in this study enable us to expand and control the differentiation status of HFSCs, providing a platform for understanding the mechanisms underlying the maintenance of the cyclical HF regenerative potential of HFSCs, lineage determination, and hierarchical relationship of multiple types of HFSCs. Our study also demonstrated the potential of HFSCs and their culture system to achieve successful HF regenerative therapy, which will be a first-in-human trial of organ regenerative medicine.

## Materials and methods

### Animals

Experiment used C57BL/6N (Japan SLC Inc.), C57BL/6-Tg(CAG-EGFP) (Japan SLC Inc.), BALB/cSlc-nu/nu (Japan SLC Inc.), and Lgr5-EGFP-IRES-creERT2 mice (Jackson Laboratory). The animals were housed in environmentally controlled rooms, and all the experimental procedures using animals were approved by the Institutional Animal Care and Use Committee of RIKEN Kobe Branch and performed in accordance with the relevant guidelines and regulations.

### Isolation of epithelial cells from adult vibrissae and back skin

HF epithelial cells from 7 to 8 weeks old vibrissae were isolated according to the method reported previously^3^. Epithelial cells from adult telogen back skin were obtained using a previously described method with minor modification^35^. Briefly, following removal of subcutaneous tissue, back skin was cut into 1.0 × 1.5 cm^2^ pieces and incubated in 0.25% trypsin (Lifetechnologies) for 50 min at 37 °C. The epidermis was separated from dermis using forceps and scalpel blades in DMEM (Lifetechnologies) containing 10% FBS (BioWest), 1% HEPES (Lifetechnologies), and 1% penn-strep (Lifetechnologies) and rinsed in fresh buffer to release basal cells into suspension and filtered through a 70 μm strainer to remove hair and cornified layers.

### Epithelial cell and dermal papilla culture

A total of 4,500 or 9,000 epithelial cells isolated from vibrissa bulge or back skin were re-suspended in 90 μl of 1% Atelocollagen solution (KOKEN) mixed with 1 × DMEM (Lifetechnologies), 10 mM HEPES (DOJINDO), and 10 mM NaHCO^3^ (Wako), respectively. After solidified of Atelocollagen, cells were cultured in 3 ml of culture medium (Supplementary Table 1) and incubated at 37 °C, 5% CO^2^. For passage experiment, cells were extracted from Atelocollagen by incubation into 100U/ml Collagenase I (Worthington) in culture medium and subsequent in 0.05% Trypsin (Lifetechnologies) for 60–90 min and 15–25 min at 37 °C. Cells were passed at 6 days in culture at a density of 9,000 cells in 90 μl of 1% Atelocollagen. Culture for dermal papilla was performed according to the method reported previously^3^. For functional analysis of TN-C, recombinant TN-C protein (R&D systems) and Neutralizing TN-C antibody (R&D systems) were added to the culture medium at the concentration of 3 or 5 mg/ml and 10 μg/ml, respectively.

### Quantification of spheroid diameter

The diameter of spheroids was measured using Cell3iMager duos (SCREEN Holdings) and calculated on Microsoft Excel.

### Flow cytometry

Single‐cell suspensions were prepared from murine vibrissa bulge, back skin, or from cultured cells as described above. Cells were rinsed once with FACS buffer composed of D-PBS (nacalai tesque), 0.2 mM EDTA (Wako), and 0.05% of BSA (sigma) and stained with antibodies for 15 min at 4 °C (Supplementary Table 2). After two wash with FACS buffer, cells were analyzed and sorted using a BD FACSAria III. Data were analyzed using FACSDiva (BD biosciences) or Weasel version 3.5.

### qPCR

RNA was isolated using the RNeasy Plus Micro Kit (Qiagen), and reverse transcribed using SuperScript VILO (LifeTechnologies) for cDNA synthesis. Real-time qPCR was performed on the Applied Biosystems QuantStudio 12 K Flex (Life Technologies) using SYBR Premix Ex Taq II (TaKaRa Bio). The data were normalized to β-actin expression. The primer pairs used for real-time qPCR are listed in Supplementary Table 3.

### Immunohistochemistry

Immunohistochemical analyses on spheroid and skin tissue were performed according to the method reported previously with minor modification^36^. Fluorescent immunohistochemistry was performed of frozen sections (10 and 50 μm) and paraffin sections (10 μm). Paraffin sections were incubated in 1 mM EDTA pH8.0 or Target Retrieval Solution pH9.0 (DAKO) for 40 min at 95 °C for antigen retrieval. All fluorescence microscopy images were obtained with an LSM 780 and LSM 880 confocal microscope (Carl Zeiss) or an Axio Scan.Z1 (Carl Zeiss). The antibodies used for immunohistochemistry are listed in Supplementary Table 4.

### Microarray analysis

Total RNA was extracted from cultured cells using the RNeasy Plus micro Kit (Qiagen) followed by synthesis of biotinylated-cRNA using GeneChip 3′ IVT PLUS Reagent Kit (Thermo Fisher Science). Labeled cRNA was hybridized to the Mouse Genome 430 2.0 Array (Affymetrix). Data were analyzed with Affymetrix GeneChip Command Console Software (Affymetrix) and Transcriptome Analysis Console (Appliedbiosystems). Heat map for selected transcriptional factors was generated using Heatmapper^37^. Data set have been deposited in the Gene Expression Omnibus (GEO) database under accession codes GSE160632.

### Reconstitution and transplantation of bioengineered HF germ

Reconstitution of bioengineered organ germ by the organ germ method and its transplantation were performed according to the method reported previously^3^. For long-term hair cycle analysis, all transplanted sites were recorded and analyzed 2–3 times a week for up to 6 months using a SteREO Lumar V12 and AxioCam fluorescent stereoscopic microscope system (Carl Zeiss, Oberkochen, Germany).

### Electron microscopy

Electron microscopic analyses were performed as described in previous study^3^.

### Statistical analyses

Student’s t-test was used to calculate P values on Microsoft Excel, with two-tailed tests.

## Supplementary information

Supplementary Information.

## References

[1] Nakao K (2007). The development of a bioengineered organ germ method. Nat. Methods.

[2] Ikeda E (2009). Fully functional bioengineered tooth replacement as an organ replacement therapy. Proc. Natl. Acad. Sci. USA.

[3] Toyoshima KE (2012). Fully functional hair follicle regeneration through the rearrangement of stem cells and their niches. Nat. Commun..

[4] Hirayama M (2013). Functional lacrimal gland regeneration by transplantation of a bioengineered organ germ. Nat. Commun..

[5] Ogawa M (2013). Functional salivary gland regeneration by transplantation of a bioengineered organ germ. Nat. Commun..

[6] Tanaka J (2018). Generation of orthotopically functional salivary gland from embryonic stem cells. Nat. Commun..

[7] Drost J, Clevers H (2017). Translational applications of adult stem cell-derived organoids. Development.

[8] Greco V (2009). A two-step mechanism for stem cell activation during hair regeneration. Cell Stem Cell.

[9] Jaks V (2008). Lgr5 marks cycling, yet long-lived, hair follicle stem cells. Nat. Genet..

[10] Snippert HJ (2010). Lgr6 marks stem cells in the hair follicle that generate all cell lineages of the skin. Science.

[11] Jensen KB (2009). Lrig1 expression defines a distinct multipotent stem cell population in mammalian epidermis. Cell Stem Cell.

[12] Horsley V (2006). Blimp1 defines a progenitor population that governs cellular input to the sebaceous gland. Cell.

[13] Ito M (2007). Wnt-dependent de novo hair follicle regeneration in adult mouse skin after wounding. Nature.

[14] Joost S (2016). Single-cell transcriptomics reveals that differentiation and spatial signatures shape epidermal and hair follicle heterogeneity. Cell Syst..

[15] Lu CP, Polak L, Keyes BE, Fuchs E (2016). Spatiotemporal antagonism in mesenchymal-epithelial signaling in sweat versus hair fate decision. Science.

[16] Tripurani SK (2018). Suppression of Wnt/beta-catenin signaling by EGF receptor is required for hair follicle development. Mol. Biol. Cell.

[17] Merrill BJ, Gat U, DasGupta R, Fuchs E (2001). Tcf3 and Lef1 regulate lineage differentiation of multipotent stem cells in skin. Genes Dev..

[18] Niemann C, Owens DM, Hulsken J, Birchmeier W, Watt FM (2002). Expression of DeltaNLef1 in mouse epidermis results in differentiation of hair follicles into squamous epidermal cysts and formation of skin tumours. Development.

[19] Estrach S, Ambler CA, Lo Celso C, Hozumi K, Watt FM (2006). Jagged 1 is a beta-catenin target gene required for ectopic hair follicle formation in adult epidermis. Development.

[20] Pan Y (2004). gamma-secretase functions through Notch signaling to maintain skin appendages but is not required for their patterning or initial morphogenesis. Dev. Cell.

[21] Chacon-Martinez CA, Klose M, Niemann C, Glauche I, Wickstrom SA (2017). Hair follicle stem cell cultures reveal self-organizing plasticity of stem cells and their progeny. EMBO J..

[22] Blanpain C, Lowry WE, Geoghegan A, Polak L, Fuchs E (2004). Self-renewal, multipotency, and the existence of two cell populations within an epithelial stem cell niche. Cell.

[23] Sato T (2009). Single Lgr5 stem cells build crypt-villus structures in vitro without a mesenchymal niche. Nature.

[24] Matsumura H (2016). Hair follicle aging is driven by transepidermal elimination of stem cells via COL17A1 proteolysis. Science.

[25] Lien WH (2011). Genome-wide maps of histone modifications unwind in vivo chromatin states of the hair follicle lineage. Cell Stem Cell.

[26] Zhang P, Andrianakos R, Yang Y, Liu C, Lu W (2010). Kruppel-like factor 4 (Klf4) prevents embryonic stem (ES) cell differentiation by regulating Nanog gene expression. J. Biol. Chem..

[27] Li J (2012). Expression of Kruppel-like factor KLF4 in mouse hair follicle stem cells contributes to cutaneous wound healing. PLoS ONE.

[28] Watt FM, Hogan BL (2000). Out of Eden: stem cells and their niches. Science.

[29] Fujiwara H (2011). The basement membrane of hair follicle stem cells is a muscle cell niche. Cell.

[30] Tucker RP, Ferralli J, Schittny JC, Chiquet-Ehrismann R (2013). Tenascin-C and tenascin-W in whisker follicle stem cell niches: possible roles in regulating stem cell proliferation and migration. J. Cell Sci..

[31] Petersson M (2011). TCF/Lef1 activity controls establishment of diverse stem and progenitor cell compartments in mouse epidermis. EMBO J..

[32] Zhang YV, Cheong J, Ciapurin N, McDermitt DJ, Tumbar T (2009). Distinct self-renewal and differentiation phases in the niche of infrequently dividing hair follicle stem cells. Cell Stem Cell.

[33] Midwood KS, Chiquet M, Tucker RP, Orend G (2016). Tenascin-C at a glance. J. Cell Sci..

[34] Fu H (2017). Tenascin-C is a major component of the fibrogenic niche in kidney fibrosis. J. Am. Soc. Nephrol..

[35] Morris RJ (2004). Capturing and profiling adult hair follicle stem cells. Nat. Biotechnol..

[36] Takagi R (2016). Bioengineering a 3D integumentary organ system from iPS cells using an in vivo transplantation model. Sci. Adv..

[37] Babicki S (2016). Heatmapper: web-enabled heat mapping for all. Nucleic Acids Res..

