# Supplementary material for: Expansion and characterization of epithelial stem cells with potential for cyclical hair regeneration
Source: Sci Rep. 2021 Feb 10;11:1173. doi: 10.1038/s41598-020-80624-3 (PMC7876088; doi:10.1038/s41598-020-80624-3)
Supplement: Supplementary file 1 — Supplementary Information. [file 41598_2020_80624_MOESM1_ESM.pdf]

Expansion and characterization of epithelial stem cells with organ-inductive potential for long-term cyclical hair regeneration

Authors:

Makoto Takeo[1, 7], Kyosuke Asakawa[1, 7], Miho Ogawa[1, 2], JingJing Tong[3], Tarou Irié[4], Masayuki Yanagisawa[5], Akio Sato[6] and Takashi Tsuji[1, 2, 3, 5, 6] *

Affiliations:

[1] Laboratory for Organ Regeneration, RIKEN Center for Developmental Biology (CDB) and RIKEN Center for Biosystems Dynamics Research (BDR), Hyogo, 650-0047, Japan

[2] Organ Technologies Inc., Tokyo, 101-0048, Japan

[3] Department of Bioscience, Graduate School of Science and Technology, Kwansei-Gakuin University, Hyogo, 669-1337, Japan

[4] Division of Anatomical and Cellular Pathology, Department of Pathology, Iwate Medical University, Iwate 028-3694, Japan

[5] Department of Plastic and Aesthetic Surgery, School of Medicine, Kitasato University, Kanagawa 252-0375, Japan

[6] Department of Plastic and Reconstructive Surgery, School of Medicine, Keio University, Tokyo 160-8582, Japan

[7] These authors contributed equally to this work.

*Correspondence to: takashi.tsuji@riken.jp

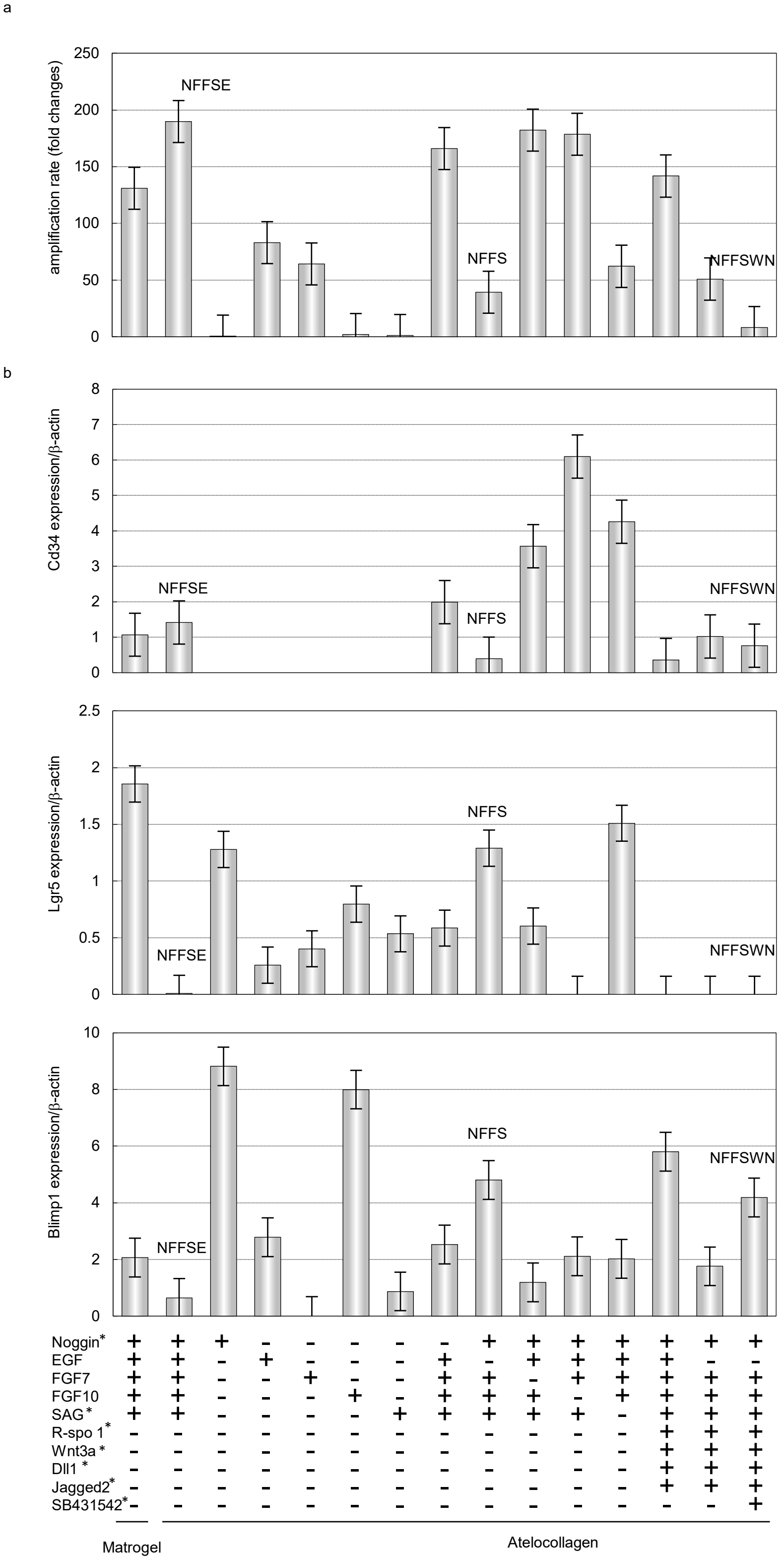
a
amplification rate (fold changes)
250
200
150
100
50
0
NFFSE
NFFS
NFFSWN
b
Cd34 expression/β-actin
8
7
6
5
4
3
2
1
0
NFFSE
NFFS
NFFSWN
Lgr5 expression/β-actin
2.5
2
1.5
1
0.5
0
NFFS
NFFSE
NFFSWN
Blimp1 expression/β-actin
10
8
6
4
2
0
NFFS
NFFSWN
NFFSE
Noggin* + + + - - - - - + + + + + + +
EGF + + - + - - - + - + + + + - -
FGF7 + + - - + - - + + - + + + + +
FGF10 + + - - - + - + + + - + + + +
SAG* + + - - - - + + + + + - + + +
R-spo 1* - - - - - - - - - - - - + + +
Wnt3a* - - - - - - - - - - - - + + +
Dll1* - - - - - - - - - - - - + + +
Jagged2* - - - - - - - - - - - - + + +
SB431542* - - - - - - - - - - - - - - +
Matrogel
Atelocollagen

Supplementary Fig. S1. Screening of culture conditions based on the amplification rate and gene expression

(a) Amplification rate of bulge epithelial cells isolated from adult mouse vibrissa cultured in various conditions for 6 days. **(b)** qPCR analysis of indicated markers on cultured cells. Asterisks indicate cytokines that different from previously reported culture method for HFSCs. Data are presented as the mean ± SD.

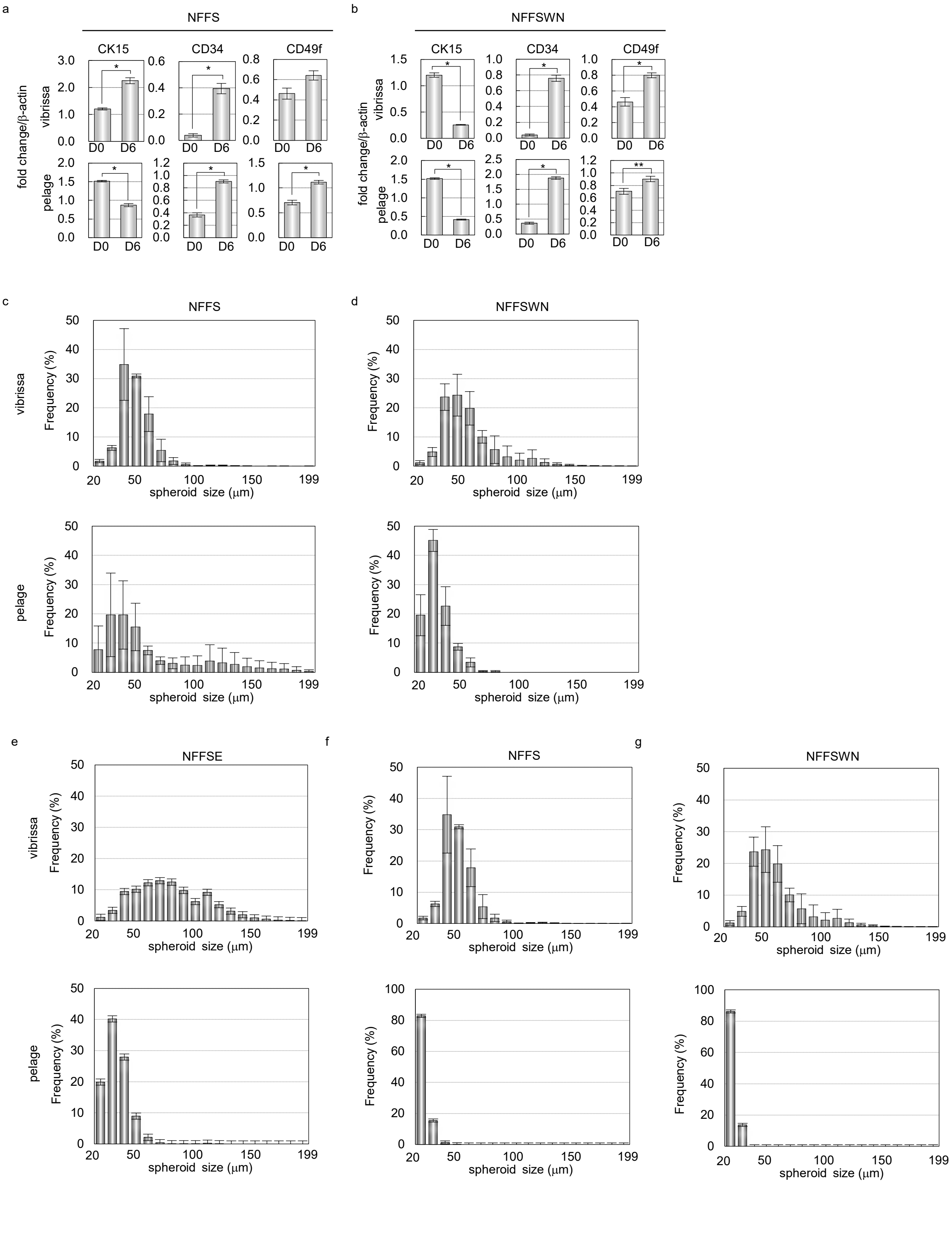
a
NFFS
CK15
CD34
CD49f
fold change/β-actin
vibrissa
pelage
D0
D6
*
b
NFFSWN
**
c
NFFS
d
NFFSWN
Frequency (%)
spheroid size (μm)
e
NFFSE
f
NFFS
g
NFFSWN

Supplementary Fig. S2. The gene expression profile and colony size were changed based on the culture conditions.

(a and b) Replicative qPCR analysis of indicated markers on freshly bulge cells and cells cultured in NFFS (a) and NFFSWN medium (b). **(c and d)** Quantification analysis of the size of spheroids cultured in NFFS (c) and NFFSWN medium (d). **(e-g)** Quantification analysis of the size of spheroids passaged in NFFSE (e), NFFS (f), and NFFSWN medium (g). Frequency was calculated from 1,000 spheroids in each condition. Data are presented as the mean ± SD. *$p < 0.001$; **$p < 0.005$.

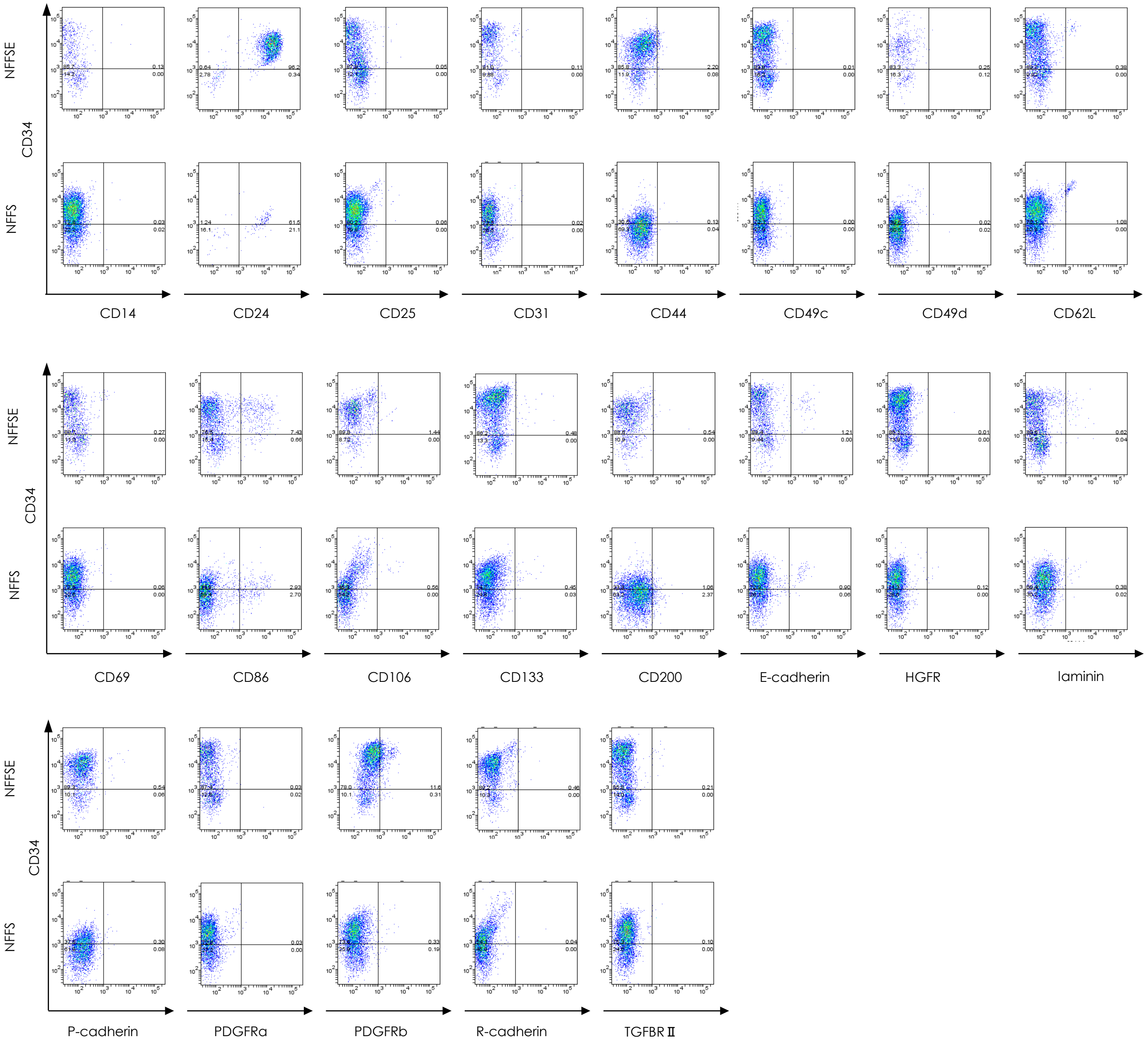
NFFSE
NFFS
CD34
CD14
CD24
CD25
CD31
CD44
CD49c
CD49d
CD62L
CD69
CD86
CD106
CD133
CD200
E-cadherin
HGFR
laminin
P-cadherin
PDGFRa
PDGFRb
R-cadherin
TGFBR Ⅱ

Supplementary Fig. S3. Comparison of cell surface marker expression among cells cultured in NFFSE and NFFS medium

Epithelial cells were isolated from the bulge region of adult mouse vibrissae and cultured in either NFFSE or NFFS medium for 6 days. The expression of the indicated cell surface markers was analyzed by FACS.

a

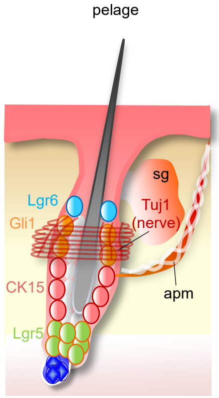
pelage
sg
Lgr6
Tuj1
(nerve)
Gli1
CK15
apm
Lgr5

b

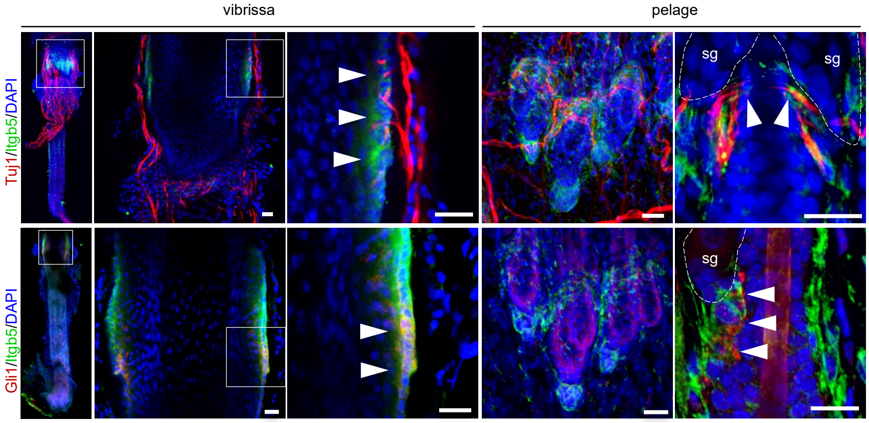
vibrissa
pelage
Tuj1/Itgb5/DAPI
Gli1/Itgb5/DAPI
sg
sg
sg

Supplementary Fig. S4. Spatial relationship between Itgb5+ cells and previously reported upper bulge markers

Schematic illustration of upper bulge markers. **(b)** Immunofluorescence of the indicated markers on native mouse vibrissa and pelage HF. Middle and right panels of vibrissa indicate a high magnification of the boxed area in the left and middle panels. Arrowheads indicate the co-expression of the indicated markers. sg, sebaceous gland. Scale bars, 20 μm.

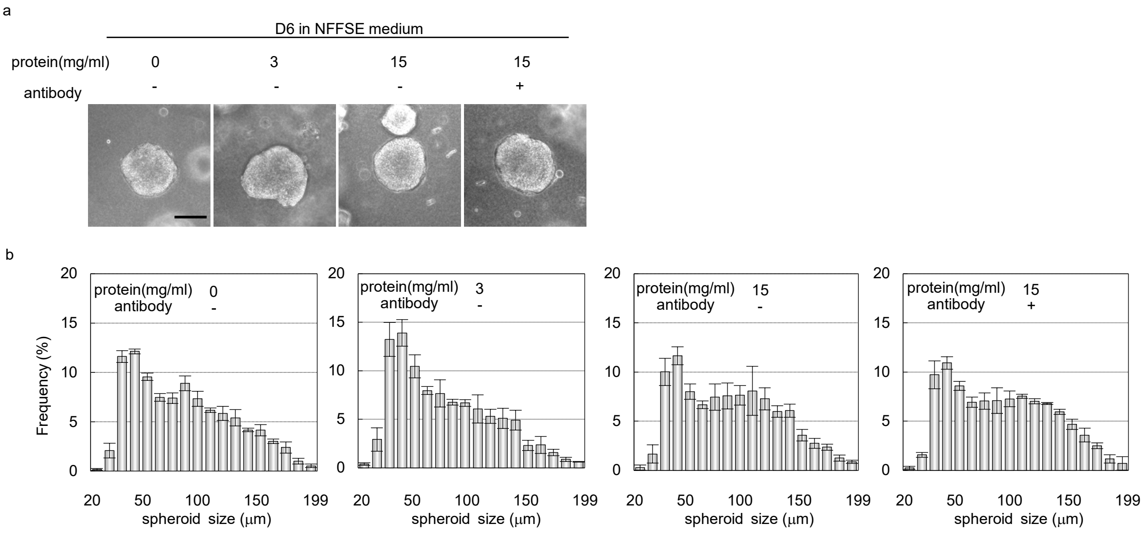
a
D6 in NFFSE medium
protein(mg/ml) 0 3 15 15
antibody - - - +
b
protein(mg/ml) 0
antibody -
protein(mg/ml) 3
antibody -
protein(mg/ml) 15
antibody -
protein(mg/ml) 15
antibody +
Frequency (%)
spheroid size (μm)

Supplementary Fig. S5. *In vitro* functional analysis of TN-C on CD34+/CD49f+/Itgβ5+ cells

(a) Phase contrast images of spheroids cultured for 6 says in NFFS with recombinant TN-C protein and anti-TN-C neutralizing antibody as indicated. (**b)** Quantification analysis of the size of spheroids cultured in indicated conditions. Frequency was calculated from 1,000 spheroids in each condition. Data are presented as the mean ± SD. Scale bars, 20 μm.

Table S1. Media compositions.

a. NFFS medium

REAGENTS	SOURCE	IDENTIFIRE	CONCENTRATION
Advanced DMEM/F12	Gibco	12634010	-
GlutaMax Supplement	Gibco	35050061	1x
HEPES	Gibco	15630-080	1x
N2 Supplement	Gibco	17502001	1x
B-27 Supplement	GIbco	17504044	1x
Murine Noggin	PEPRO TECH	250-38	50ng/ml
Recombinant Mouse FGF7	R&D Systems	5028-KG	50ng/ml
Recombinant Mouse FGF10	R&D Systems	6224-FG-025	50ng/ml
SAG	Enzo Life Science	ALX-270-426	50ng/ml

b. NFFSE medium

REAGENTS	SOURCE	IDENTIFIRE	CONCENTRATION
NFFS medium			-
Murine EGF	PEPRO TECH	315-09	50ng/ml

c. NFFSNW medium

REAGENTS	SOURCE	IDENTIFIRE	CONCENTRATION
NFFS medium			-
Recombinant Mouse Wnt-3a	R&D Systems	1324-WN-010	50ng/ml
Recombinant Mouse R-spondin1	R&D Systems	3474-RS-050	1μg/ml
Recombinant Mouse Dll1	R&D Systems	550-20531	1μg/ml
Recombinant Mouse Jagged-2	R&D Systems	4748-JG-050	1μg/ml
SB431542	TOCRIS	1614	50ng/ml

Table S2. Antibodies list for FACS analysis.

Antibodies	HOST	DILUTION	SOURCE	IDENTIFIRE
FITC anti-mouse CD14	Rat	1:50	BD PharMingen	09471D
Anti-CD24	Rat	1:50	abcam	ab64064
FITC Anti-mouse CD25	Rat	1:50	BD PharMingen	01094D
Anti-CD31	Rabbit	1:50	abcam	ab28364
CD34 Monoclonal Antibody, eFluor 660	Rat	1:50	eBioscience	4329945

Anti-mouse CD44	Rat	1:50	BD PharMingen	553724
Anti-CD49c	Mouse	1:50	BD Transduction Laboratories	611044
Anti-mouse CD49d	Rat	1:50	BioLegend	103708
PE Anti-human/mouse CD49f antibody	Rat	1:50	BioLegend	313612
FITC Anti-mouse CD62L	Rat	1:50	BD PharMingen	01264D
FITC Anti-mouse CD69	Hamster	1:50	BD PharMingen	01504D
Anti-mouse CD86	Rat	1:50	BD PharMingen	09270D
Anti-mouse CD106	Rat	1:50	BD PharMingen	550547
Anti-CD133	Rabbit	1:50	abcam	ab16518
Anti-CD200	Rat	1:50	abcam	ab33734
FITC Anti-E-Cadherin	Mouse	1:50	BD Biosciences	612130
Anti-mHGF Receptor	Goat	1:50	R&D systems	AF527
Anti-integrin beta 5	Sheep	1:50	R&D systems	AF8035
Anti-Laminin antibody	Rabbit	1:50	abcam	ab11575
Anti-P-Cadherin	Rat	1:50	R&D systems	MAB761
Anti-PDGF Receptor alpha	Rabbit	1:50	abcam	ab61219
Anti-PDGF Receptor beta	Rabbit	1:50	abcam	ab32570
Anti-TGF beta Receptor II	Rabbit	1:50	abcam	abcam
Anti-Rat IgG, Alex Fluor 488	Donkey	1:1,000	Invitrogen	A21208
Anti-Rabbit IgG, Alex Fluor 488	Donkey	1:1,000	Invitrogen	A21206
Anti-Mouse IgG, Alex Fluor 488	Donkey	1:1,000	Invitrogen	A21202
BV421 Anti-Sheep IgG	Donkey	1:1,00	Jackson ImmunoResearch	713-675-147

Table S3. Primers list for real-time qPCR.

Primers	NCBI Gene ID	sequence
Actb	11461	tgacaggatgcagaaggaga
		gctggaaggtggacagtgag
CD34	12490	aagaccacaccagccatctc
		ggggaagtctgtggttgtga
CD49f	16403	aaggcctctctcgttcttcg
		gggagcttgatattctgagcag
CK15	16665	acaacatgctgctggacatc
		gcttcctcccagggatacttc
Lhx2	16870	cctactacaacggcgtgggcactgt
		gtcacgatccaggtgttcagcatcg
Sox9	20682	cgtggacatcggtgaactga
		ggtggcaagtattggtcaaactc
Col17a1	12821	gagggaacaaaggagagaaagg
		aagaaccggtcccagatacttg
Lgr5	14160	tccaacctcagcgtcttcac
		ccttgggaatgtgtgtcaaagc
Lrig1	16206	ggtccctctatccaagcaacc
		acgtaggagaaggctttaggtc
Blimp1	12142	atgtctgtgccaagacgttc
		ccaagtagtgtttctgcaggtg

Table S4. Antibodies list for immunohistochemistry.

Antibodies	HOST	DILUTION	SOURCE	IDENTIFIRE
Anti-Cytokeratin 15	Chicken	1:100	BioLegend	833904
Anti-CD34	Rat	1:50	eBioscience	14-0341-82
Anti-CD49f	Rat	1:100	BioLegend	313613
Anti-GFP	Chicken	1:100	abcam	ab13970
Anti-Blimp1	Mouse	1:50	Santa Cruz	sc-66015
Anti-integrin beta 5	Rabbit	1:50	abcam	ab15459
Anti-Tenascin C	Rabbit	1:50	abcam	ab108930
Anti-Tenascin N	Rabbit	1:50	LsBio	LS-C160093
Anti-Chicken IgY, Alexa Fluor 594	Goat	1:200	Invitrogen	ab150172
Anti-Rat IgG, Alex Fluor 488	Donkey	1:200	Invitrogen	A21208
Anti-Mouse IgG, Alex Fluor 488	Donkey	1:200	Invitrogen	A21202
Anti-Rabbit IgG, Alex Fluor 488	Donkey	1:200	Invitrogen	A21206
